# Modeling of Mitochondria Bioenergetics Using a Composable Chemiosmotic Energy Transduction Rate Law: Theory and Experimental Validation

**DOI:** 10.1371/journal.pone.0014820

**Published:** 2011-09-08

**Authors:** Ivan Chang, Margit Heiske, Thierry Letellier, Douglas Wallace, Pierre Baldi

**Affiliations:** 1 Department of Biomedical Engineering, University of California Irvine, Irvine, California, United States of America; 2 Institute of Genomic Biology, University of California Irvine, Irvine, California, United States of America; 3 INSERM U688, University of Bordeaux-2, Bordeaux, France; 4 Department of Biochemistry, University of California Irvine, Irvine, California, United States of America; 5 Center for Mitochondrial and Molecular Medicine and Genetics (MAMMAG), University of California Irvine, Irvine, California, United States of America; 6 Department of Computer Science, University of California Irvine, Irvine, California, United States of America; University of South Florida, United States of America

## Abstract

Mitochondrial bioenergetic processes are central to the production of cellular energy, and a decrease in the expression or activity of enzyme complexes responsible for these processes can result in energetic deficit that correlates with many metabolic diseases and aging. Unfortunately, existing computational models of mitochondrial bioenergetics either lack relevant kinetic descriptions of the enzyme complexes, or incorporate mechanisms too specific to a particular mitochondrial system and are thus incapable of capturing the heterogeneity associated with these complexes across different systems and system states. Here we introduce a new composable rate equation, the chemiosmotic rate law, that expresses the flux of a prototypical energy transduction complex as a function of: the saturation kinetics of the electron donor and acceptor substrates; the redox transfer potential between the complex and the substrates; and the steady-state thermodynamic force-to-flux relationship of the overall electro-chemical reaction. Modeling of bioenergetics with this rate law has several advantages: (1) it minimizes the use of arbitrary free parameters while featuring biochemically relevant parameters that can be obtained through progress curves of common enzyme kinetics protocols; (2) it is modular and can adapt to various enzyme complex arrangements for both *in vivo* and *in vitro* systems via transformation of its rate and equilibrium constants; (3) it provides a clear association between the sensitivity of the parameters of the individual complexes and the sensitivity of the system's steady-state. To validate our approach, we conduct *in vitro* measurements of ETC complex I, III, and IV activities using rat heart homogenates, and construct an estimation procedure for the parameter values directly from these measurements. In addition, we show the theoretical connections of our approach to the existing models, and compare the predictive accuracy of the rate law with our experimentally fitted parameters to those of existing models. Finally, we present a complete perturbation study of these parameters to reveal how they can significantly and differentially influence global flux and operational thresholds, suggesting that this modeling approach could help enable the comparative analysis of mitochondria from different systems and pathological states. The procedures and results are available in Mathematica notebooks at http://www.igb.uci.edu/tools/sb/mitochondria-modeling.html.

## Introduction

Throughout the mitochondria inner membrane are many energy-transducing protein complexes that help transform the chemical energy from the cell's metabolic intake into various useful forms of energy for the cell. Some of these complexes use the free-energy extracted froms reduction-oxidation (redox) reactions to transport proton across the membrane and establish a proton gradient, while others use this proton gradient in combination with the membrane potential, the proton-motive-force (*pmf*), to drive otherwise energetically unfavorable processes such as ATP synthesis and assorted transporters of other ions and/or molecules. This use of the *pmf* as an intermediate driving force in the overall conversion of energy is the essence of the chemiosmotic theory [1], and the flow of energy between these chemiosmotic complexes constitutes the core of mitochondrial bioenergetics [2]. The chemiosmotic complexes consist of complex I, III, IV, and V of the oxidative phosphorlyation (OXPHOS) pathway, and are partly encoded by the mitochondria DNA (mtDNA). Genetic variation or mutations in the mtDNA can alter the protein structures of the complexes, which can then affect their functional output, the bioenergetics of the system, and ultimately the health of the organism. In particular, a mtDNA mutation in a polypeptide of an electron-transport-chain (ETC) complex may cause its enzyme machinery to become less efficient in its energy transduction. An increase in the slippage of complex I and III of the ETC [3]–[5] can lead to an increase in the production of the respiration byproduct, reactive oxygen species (ROS), which can further damage the mtDNA and create a vicious feed-forward loop of energetic decline. When the total damage to the OXPHOS surpasses a functional threshold whereby it can no longer fulfill the energetic requirements of the cell, the cell may undergo apoptosis (programmed cell death) to remove itself from the population. The consequence of such an energetic decline is potentially grave, as over time, when enough cells are lost, the organism would begin to lose the functions of its organs, which might be manifested as either the normal progression of aging, or more seriously as the onset of major metabolic and degenerative diseases such as diabetes, Alzheimer, Parkinson, as well as cancer [6], [7].

Interest in the roles that mitochondria play in mammalian health and disease has grown markedly over the past two decades, resulting in an abundance of genetic [7]–[10], structural [11], [12], biochemical [13]–[15], and pathological [16], [17] studies on mitochondria systems. An effective integration of the heterogeneous data coming from these studies is the main focus of the emerging field of systems biology [18]. However, the development of one of its key ingredients– the kinetic modeling of mitochondria bioenergetics–has not kept pace with the rest of the field and could potentially become a bottleneck.

Models of mitochondrial bioenergetics range from the top-level network-constraint variety [19], all the way down to the low-level molecular dynamics simulations [20]. Nevertheless, ODE based deterministic approaches still provide the best balance of dynamic descriptions and computational tractability across several length and time scales [21]. Of the deterministic models, the simplest and most direct approach is to approximate the respiration flux through the whole mitochondria by using a single empirical oxygen consumption equation [22], [23]. To introduce a general thermo-kinetic approach, Jin and Bethke describe a respiration rate law that encompasses the overall electro-chemical reaction of the respiratory chain, and is applicable to both mitochondrial and bacterial respiration models [24], [25]. Single equation approaches such as these, allow easy assimilation of cellular respiration into higher scale models, but lack the level of detail required to understand the contributions from the individual components.

At the next level of detail, the respiration process is divided into its principle components in the OXPHOS pathway, each with its own kinetic description. There are currently three main components-based OXPHOS models that serve as the basis for other larger and more extensive physiological models: the Yugi and Tomita model [26], [27], the Korzeniewski model [28], and the Beard model [29]–[32]. These approaches have been used in the studies of *in vivo* cardiac energy metabolism [33](Beard model) [34] (Korzeniewski model), dynamic OXPHOS respiration simulation [35](Beard model), volume dynamics of mitochondrial bioenergetics [36](Beard model), mitochondrial fatty acid 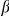 -oxidation network [37](Yugi and Tomita model), the modeling of the ETC in purple non-sulfur bacteria [38](Korzeniewski model), etc. Although the three OXPHOS models have been useful in studying several aspects of bioenergetics and physiology, they are limited by their choices of mechanism schemes. In particular, the Yugi model assembles a large array of detailed kinetic descriptions derived from specific enzyme binding mechanisms in past literature, e.g. the Ping-Pong bi bi mechanism of complex I [39] etc., but treats them as separate and independent “reactors” that do not incorporate the thermodynamic constraints necessary to characterize the dependence of their energy transduction processes to the chemiosmotic forces of the system. The Korzeniewski model primarily uses empirical data-driven relationships in both its reaction equations and systems properties, and it incorporates linear thermodynamic constraints on its OXPHOS components based on their free-energy profiles. However, the validity of its linear approximation is limited to near-equilibrium conditions. The Beard model inherits many components from the Korzeniewski model, but it extends the thermodynamic constraints to non-linear and far-from-equilibrium regions, and it explicitly treats the membrane potential and proton gradient of the system as separate state variables. However, the reaction rate equations in the Beard model lack detailed “kinetic descriptions of enzyme activity” [35]. In other words, these reaction rate equations do not intrinsically account for the kinetic properties of the ETC complexes since they were not derived in view of the internal mechanisms of the complexes. Instead, kinetic parameters are incorporated mainly through phenomenological control factors, which are introduced to compensate for specific modulations shown in experimental data sets.

The internal mechanisms of the complexes have been modeled with explicit elementary reaction steps to track intermediate metabolic species or reaction byproducts such as the generation of ROS [3], [20]. However, at this scale, model validation becomes exceedingly difficult with large uncertainty as the individual reaction rates are not observable with the experimental technology currently available. For models that do incorporate detailed mechanistic schemes, there is the additional danger of overfitting in that mitochondria across different systems display a high degree of variability in their components [15], and consequently if a detailed model is based on a particular system, it may not generalize well to other systems. In addition, kinetic properties that are determined under a certain set of conditions (e.g. an *in vitro* laboratory setup) may not be transformable into another set of condtions (e.g. *in vivo* system). Thus, if a model of mitochondrial bioenergetics is to be both extensive and flexible, it must be able to adapt to the mechanism of a particular system, and be applicable to various system conditions. Furthermore, the dynamics of a system depend on the system's component descriptions, yet a system is often more than just the sum of its components. Thus, to establish a functional relationship between the dynamics of a system and its components, it is also necessary to allow for potential unobserved properties to emerge from the synergistic network interactions that vary from system to system. Only then can a model effectively capture the system's response to a perturbation across multiple scales.

In this paper, we present a new modeling approach for mitochondrial bioenergetics that addresses the problems of cross-mechanism adaptability, cross-conditional applicability, and cross-scale analysis through a novel composable kinetic rate equation that we term the chemiosmotic rate law. The rate law incorporates three configurable modulating factors that, via steady-state and rapid-equilibrium approximations, separately encapsulate the binding kinetics, the redox transfer potential, and the non-linear thermodynamic force-to-flux relationship of a prototypical energy transduction complex in the OXPHOS pathway. The separation of the factors allows one to selectively configure the mechanistic scheme of the complex, while the six biochemically relevant kinetic parameters of the rate law allow one to completely specify its kinetic properties at a particular reference condition. The kinetic parameters are estimated from data obtained experimentally using simple extensions to the standardized *in vitro* assay protocols for the various ETC complexes in the OXPHOS pathway. The resulting *in vitro* reference dynamics can be transformed into dynamics of an *in vivo* system through changes in the system's constituent thermodynamic forces, the reaction equilibrium constant, and the rate constants in a systematic way. One particularly useful type of transformation is a “slippage”, or a loss in the thermodynamic efficiency of the complex's energy transduction process, which can be used to generally express a perturbation on the complex's reaction rate.

To validate the rate law, we conduct six parallel sets of assays on rat muscle homogenates, two for each complex. For each complex, we obtain kinetic parameter estimates from one of the two sets of assays, and then compare the progress curve predicted by our model to the experimental progress curve from the other set of assays. In addition, we formally establish the theoretical connections between our approach and other models, and compare their predictive accuracies. Finally, by using the optimized parameters for complex I, III, and IV, we perform a complete perturbation study of these parameters to reveal how they can significantly and differentially influence global flux and operational thresholds, suggesting that this modeling approach could help enable the comparative analysis of mitochondria from different systems and pathological states.

## Methods

In this section, we first describe the derivation of the new chemiosmotic rate law based on the common properties of the ETC complexes. We then describe the experimental protocols that can be used to obtain the various biochemical parameters appearing in the rate law.

### ETC Complex Rate Law Derivation

#### ETC Complexes and their Common Properties

**Figure 1:**
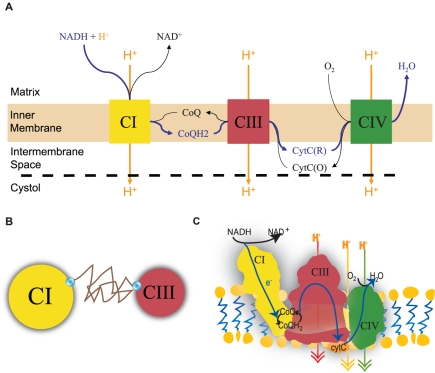
Electron Transport Chain (ETC). **A.** Standard model of ETC where electrons are shuttled from one complex to the next through diffusion of the intermediate electron carriers (ECs). **B.** Random walk diffusion of ECs in the random collision model. **C.** Supercomplex model of the ETC. The complexes of the ETC are assembled together into a super structure which reduces the diffusion time of the intermediate ECs by providing a direct conduit for electron transfer.

The ETC consists of four integral membrane protein complexes that facilitate a sequence of electron transfer steps: (1) Electrons from citric-acid cycle products 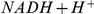 and 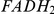 enter the complex I (*NADH-ubiquinone oxidoreductase*) and complex II (*succinate-ubiquinone oxidoreductase*) respectively; (2) both complexes then catalyze the reduction-oxidation transfer of electrons to 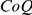 (ubiquinone) to produce 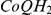 (ubiquinol); (3) subsequently, 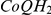 is bound by complex III (*ubiquinol-cytochrome c oxidoreductase*), which transfers the electrons to cytochrome c; and (4) cytochrome c binds to complex IV (*cytochrome c-* 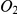 *oxidoreductase*), which transfers the electrons to oxygen, the final electron acceptor [40] (Figure 1A). The ETC complexes can either move about freely by lateral diffusion in the plane of the membrane (Figure 1B), or alternatively, the complexes can form aggregates or supercomplexes, ranging from small clusters of a few complexes to a complete ETC assembly [11] (Figure 1C), to enable direct electron channeling between the complexes.

Each type of ETC complex is structurally unique, has diverse catalytic and binding rates, and responds to different inhibitors. Much of the detailed mechanisms of each complex remain to be determined. Nevertheless, all ETC complexes and their supercomplex assemblies share the same general characteristic of binding to both a donor and an acceptor electron carrier (EC) species, and facilitating the flow of electrons through the complex by a series of intermediate internal redox steps [2]. In addition, all complexes except for complex II capture the energy from such an electron flow and couple it to the drive of protons against their electrochemical gradient across the mitochondria inner membrane, much like how a windpump (combination of a windmill and a water pump) captures the energy generated by air flow to move water against gravity. Both are examples of an energy transduction process in which free-energy from an energetically favorable flow of one type of particle drives an energetically unfavorable flow of another type of particle through a type of coupling catalytic machine. However, as with all types of coupling catalytic machines, they can deviate from their normal coupling efficiency (slip) when damaged or when operating at an abnormal turnover rate.

#### Electron-Proton Pump (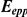) Representation

**Figure 2:**
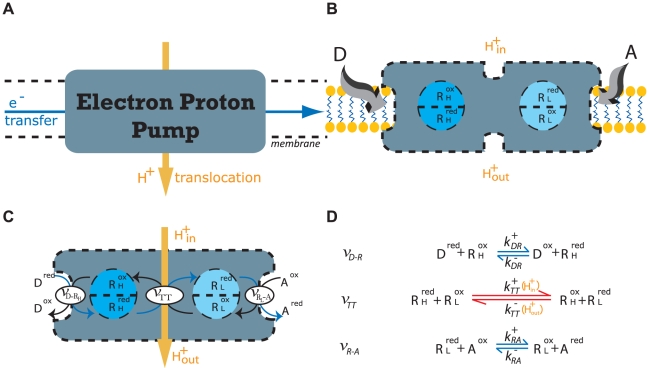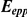 Representation of a Chemiosmotic ETC Complex. **A.** The electron-proton pump 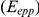 uses the free-energy captured from the electron transfer to translocate protons (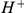) across the membrane. **B.** Internal structure of the 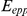 consisting of one binding/reaction site each for the donor electron carrier (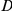) and the acceptor electron carrier (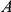), one high potential redox center (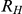), and one low potential redox center (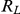). **C.** The three internal processes of the electron-proton pump: reaction 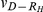 captures the transfer of electron(s) from reduced form of the donor electron carrier 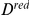 to the high potential internal redox center 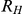; reaction 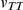 tunnels the electron(s) between 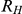 and the low potential redox center 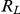 and drives the proton translocation; reaction 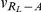 allows the exit of the electron(s) from 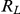 to the oxidized form of the acceptor 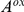. **D.** Chemical reaction equation representation of the three internal processes: the red bidirectional arrows indicate the rate limiting step, and the blue bidirectional arrows represents processes under fast equilibrium condition relative to the rate limiting step.

To provide quantitative descriptions for the ETC complexes, an abstract prototypical ETC complex model, the electron-proton pump (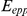) complex, is introduced based on the aforementioned general properties of the ETC complexes. Externally, this 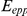 complex is embedded in a membrane that separates two compartments, and its function is to catalyze the energy transduction between an electron transfer reaction and a proton translocation reaction (Figure 2A). This overall transduction reaction is expressed by the chemical equation:
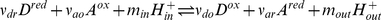
(1) where 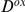 and 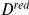 are the oxidized and reduced species of the donor-EC reactant (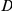), 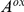 and 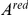 are the oxidized and reduced species of the acceptor-EC reactant (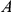), 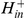 and 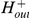 are the proton inside and outside of the membrane, while 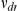, 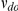, 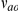, 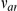, 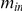 and 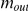 represent their stoichiometric reaction coefficients respectively. The overall chemical reaction for each of the ETC complexes is tabulated in Table 1.

**Table 1:** ETC Complex Reaction and Stoichiometry.

	Overall Reaction	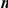	e 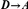	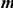
CI	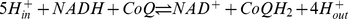	2	0	4
CIII	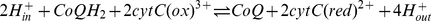	2	−1	4
CIV	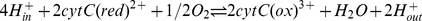	2	0	2

Assuming that the concentrations of chemical species and reactants are homogeneous inside each compartment, the dynamics of the overall reaction is governed by a set of time-dependent ordinary differential equations from the law of conservation of mass [41]. The rate of change for the chemical concentrations is directly related to the turnover rate of the reaction, expressed in terms of the reaction flux 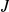, by their respective stoichiometric coefficients:
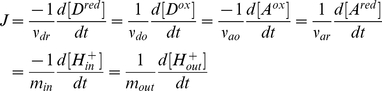
(2)


In addition, depending on the composition of 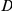 and 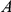, for each turn of the reaction there is 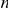 net number of electron transferred between 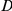 to 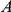, giving the implicit electron flux:
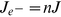
(3)


**Figure 3:**
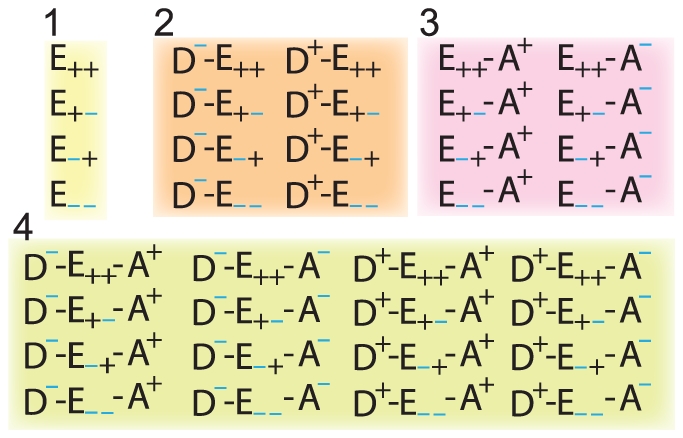
Representative Microstates. There are 36 distinct configurations of the complex that contribute to the forward or reverse turnover rate. They include: (**1**) 4 configurations of the fully unbound 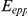 (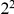 distinct states of the two internal redox-centers); (**2**) and (**3**) 2×8 configurations of both 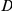 -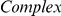 or 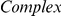-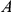 partially bound 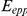 (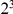 distinct states from the addition of either one bound EC); and (**4**) 16 configurations of the fully bound 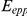 (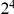 distinct states from all four binary reaction centers).

The turnover rate depends on various catalytic properties of the enzyme that the 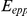 complex represents, thus a detailed quantitative description of the reaction flux 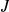 for the 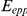 complex must take into account its internal structure and molecular mechanisms. Internally, the 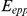 complex can be modeled using four general features: one donor EC binding site, one acceptor EC binding site, one high potential redox-center 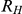, and one low potential redox-center 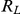 (Figure 2B). At any instant, the EC binding sites can either be occupied or unoccupied, while the bound ECs and the internal redox-centers can either be reduced or oxidized, giving rise to a total of 64 microstates (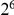 distinct configurations) of the reaction system. Of these microstates, 36 are distinct configurations of the complex that contribute to the forward or reverse turnover rate (Figure 3). Transitions between these discrete microstates arise from the infinitesimal thermal fluctuations in the system, and they can be partitioned into the following elementry reactions that make up the overall reaction: two EC substrate binding reactions that consist of the EC binding and unbinding transition events (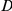 and 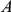 binding, Figure 2B); an electron exchange reaction between the donor EC and 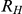 (represented by the reaction rate 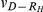); an internal electron transfer reaction between 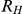 and 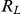 that can be coupled to the translocation of protons across the membrane (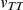); and an electron exchange reaction between 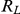 and the acceptor EC (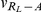) (Figure 2D). An averaging of these microstates and elementry reactions over an ensemble of 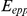 complexes provides the necessary link to the more practical macroscopic states of the system governed by the principles of thermodynamics.

#### Micro-Macroscopic Thermodynamics

The laws of thermodynamics are phenomological in nature, but they provide a convenient and powerful method of relating experimental bulk properties of a system such as pressure, volume, temperature and composition, which allows one to obtain desirable information on a system even if explicit knowledge of the interactions within the system is not available. Statistical mechanics provides the link between the quantum mechanical molecular properties and the macroscopic properties of thermodynamics by predicting an appropriate thermodynamic function of a system from its molecular structure and intermolecular forces. In regards to the reaction flux 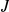, a crucial prediction derivable from statistical mechanics is how the thermodynamic forces of a system (which can also be expressed in terms of the Gibbs free energy gradient) can affect the magnitude and direction of 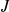 at steady-state.

Macroscopically, in a closed-system where there is no exchange of material with the outside, all chemical and physical processes eventually reach a balance such that there is no net activity in the system. At this thermodynamic equilibrium, the ratio between the concentrations (or the difference between the chemical potentials) of the products and substrates of a chemical reaction reaches a constant value defined as the equilibrium constant for that reaction. For any other ratio, there exists a disequilibrium in the chemical potentials, which provides the thermodynamic force 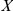 to drive the reaction towards equilibrium. By the same token, a disequilbrium from an electrostatic potential difference across a membrane also contributes to 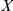. Living systems are open-systems in which materials are constantly being transported into and out of the system, driving the potentials of the various reactants and species far from their equilibrium. Analysis of non-equilibrium conditions is more intricate, but one can extend much of the equilibrium analysis to steady-states in which the system reaches a stationary condition while the input and output of the system are maintained at the same constant rate. At the steady-state, all potentials settle at a “local equilibrium” in which 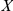 remains constant, and continually drives the reaction with a constant net flux 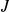.

In the following subsections, we use a combination of statistical steady-state thermodynamics, macroscopic equilibrium thermodynamics, and kinetics theories to derive a quantitative expression of the reaction flux 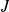 of the 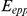 complex as a function of its thermodynamic force 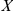 and the substrate concentrations in the reaction system. The resultant rate law provides a unification of the key attributes from the existing approaches we described [24], [26], [28], [29]. In particular, we retain in the framework of our rate law, the convenient modular approach of Jin and Bethke that partitions the different contributions to the reaction flux into separate factors that modulate on a maximum reaction velocity. The derivation proceeds first by focusing on the steady-state thermodynamics of the overall redox reaction in a post-binding ternary 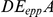 complex (*thermodynamic force function*), then coupling the net steady-state flux to the average transition rate between the internal redox centers (*redox state function*), and finally superimposing the kinetics of the binding reactions that produces the active ternary 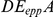 complex (*saturation function*). In addition, we extend the derivation to allow adaptation of the flux expression to different system settings by means of transformations of rate constants and/or equilibrium constants from a reference system. We then utilize the transformation framework to introduce the chemiosmotic free energy transduction mechanism of the 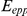 complex, as well as the slippage mechanism in the transduction process.

#### Thermodynamic Force Function 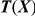


**Figure 4:**
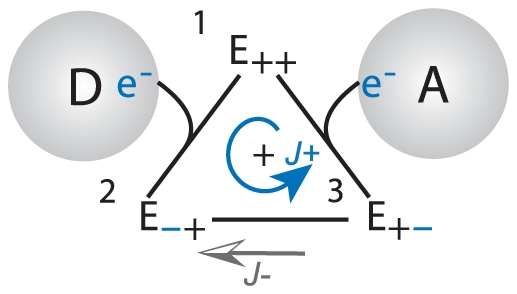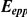 Redox Kinetic Diagram. The redox reaction of an 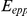 complex bound with a donor and acceptor ECs proceeds through cyclic transitions among its three representative electron states. **state 1:** 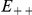 is the free state without electrons. **state 2:** 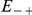 contains electron(s) obtained from the donor EC. **state 3:** 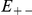 contains an electron transfered across the complex and ready for transfer to an acceptor EC. In an ensemble of 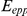
s, the number of transitions per unit time in the counter-clockwise direction constitutes the forward cycle flux 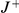, while the number of transitions per unit time in the clockwise direction constitutes the reverse cycle flux 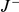.

Let us first consider the redox mechanism of the 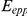 complex after it is bound with both its donor and acceptor ECs to form the active ternary 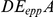 complex by using the cyclic kinetic diagram in Figure 4. The cyclic diagram is a transition map for the 16 fully bound microstates of Figure 3 condensed into three stable operational states (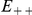, 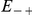, and 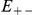), where contributions from 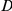 and 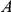 are implicit in the transfer of electron(s) to and from the 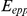 complex (transitions 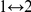 and 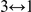), while the complex shuttles the internal transfer of electron(s) between the source and the sink (transitions 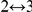). 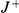 is the forward cycle flux that traverses the three states in a forward reaction sense (counter-clockwise), while 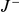 is the reverse cycle flux that travels in the opposite sense (clockwise). In a large ensemble of 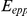 complexes, some complexes would be operating in the forward sense (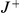), while others would be operating in the reverse sense (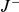), and the observed net reaction flux 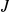 is the difference between these cycle fluxes
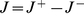
(4)


At equilibrium 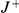 and 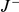 are in balance and the net flux produced is zero, whereas at a steady-state away from equilibrium, a net flux is produced by the total thermodynamic force 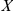 of the system. In Figure 4, this 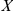 is the redox force
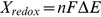
(5) where 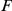 is the Faraday constant, 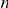 the number of electron(s) transferred, and
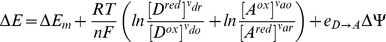
(6) is the redox potential difference for one electron between the electron carriers 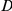 and 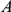. In Equation 6, 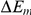 is the standard mid-point redox potential [2], 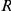 is the universal gas constant, 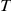 is the temperature, 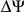 is the electrical potential difference across the membrane, while 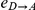 is a switch with the value of 1 if the electron is transferred from the negative N side to the positive P side of the membrane, −1 if transferred in the opposite direction, and 0 if the electron does not cross the membrane. For mitochondrial inner membrane, N is the matrix while P is the inter-membrane space.

By applying detailed balance on the individual elementry transition rates between the states in a cyclic system, a flux-to-force relationship is found that equates the steady-state ratio of the forward and reverse cyclic flux to the exponential of the thermodynamic force [41]–[43]:
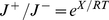
(7)


Combining Equation 7 with the definition of 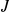 in Equation 4, one obtains the two equivalent equations
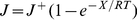
(8) and
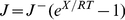
(9)in which the net reaction flux can be expressed exclusively as a function of the forward flux 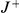 and 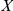, or as a function of the reverse flux 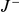 and 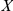. Although equivalent, we note that in the direction and limit of 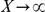, Equation 8 provides a more convenient and numerically stable expression of 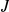, while Equation 9 is better used in the direction and limit of 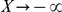. For regions in the domain of 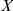 close to the equilibrium, either equation is just as good and the choice depends on whether 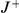 or 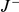 is more obtainable. Here the thermodynamic force function is introduced as:
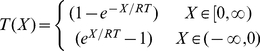
(10) which encapsulates the modulation on 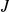 by 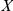, and simplifies the expression of 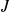 to:
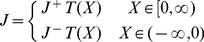
(11)


#### Redox State Function 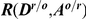


After establishing the flux-to-force relationship of the general redox reaction of the 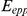 complex, the next step is to find the expressions of cycle fluxes 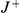 and 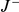. In the internal redox reactions defined earlier, the transfer-transport reaction 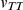 is a lump reaction of all intermediate electron transfer steps between the two outer-most redox reaction sites and also where the proton transport may occur. Therefore, comparing to 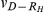 and 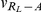, 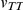 can be assumed to be the rate-limiting portion of the overall reaction, which, at the steady-state, can be coupled to the steady-state expression for 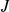.

**Figure 5:**
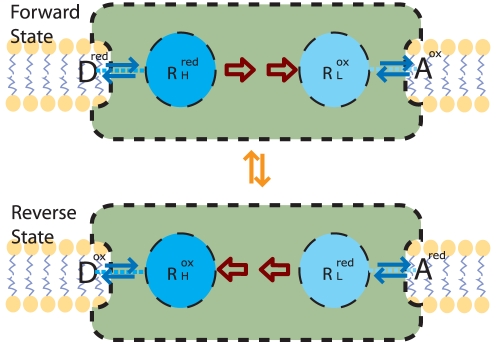
Representative Transition States. In the steady-state condition, all microscopic states of 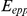 combine to form the representative forward and reverse transition states.

Microscopically, 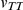 represents the transitions between the two states 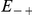 and 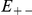 (Figure 4 and 5). Analogous to the net reaction flux (Equation 4), the net transition flux between two microstates is the difference between its forward and reverse transition rates:
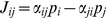
(12) where 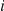 and 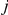 are indexes referring to the two distinct neighboring states, 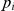 and 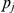 are the corresponding state probabilities, and 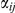 and 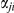 are the transition rate constants associated with the transitions from 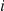 to 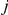 and from 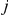 to 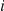 respectively. For 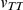, 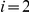 and 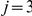 and the state probability 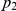 is the combined probability that 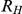 is in its reduced state 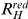 while 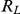 is in its oxidized state 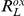, a necessary condition for the forward reaction (see Figure 5). Similarly, the state probability 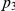 is the combined probability of 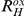 and 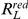, the condition necessary for the reverse reaction.

Taking into account that each 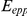 complex consists of one 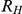 and one 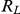, the probability of a redox-center to be in either oxidized or reduced state over an ensemble of ternary 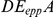 complexes is determined by dividing the concentrations in each state by the concentration 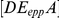:
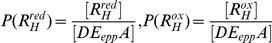


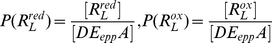
(13) which are subject to the constraints:
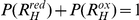


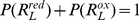
(14)


The probability for the forward state can then be taken as
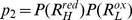
(15) and the reverse state as
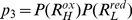
(16)


Substituting for the probabilities in the net transition flux (Equation 12) and redefine 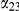 and 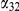 as kinetic rate coefficients 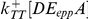 and 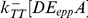, one arrives at the kinetic expression for 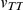:
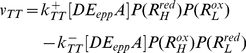
(17)


The equilibrium constant for the reaction is found by noting that 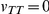 at the equilibrium:
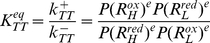
(18) where the probabilities 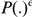 are the state probabilities at the equilibrium. Equating the rate-limiting transition flux in Equation 17 to the steady-state expression of 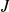 in Equation 4 gives an updated expression of Equation 11:
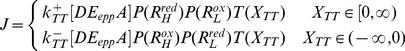
(19) where
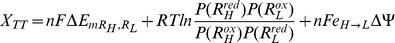
(20) is the thermodynamic force in the range of the 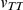 reaction. Equation 19 now completely describes the steady-state flux of the 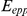 complex, but the actual state values for 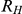 and 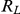 could not be measured directly since they are states within the complex. Nevertheless, a correlation between the concentrations of the external ECs and the internal redox-centers could be made such that the internal state values can be inferred from measurements of external concentrations. Since 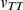 is the rate-limiting reaction, it follows that the kinetic constants for the exchange reactions 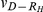 and 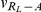 are much larger than 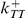 and 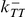, and the exchange reactions may be assumed to achieve rapid-equilibrium. As described by Jin and Bethke, with this assumption the thermodynamic forces for the two boundary exchange reactions 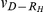 and 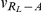 can be approximated as zero:
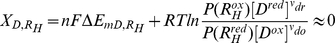


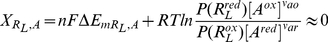
(21)


This allows one to approximate the total thermodynamic force spanning from D to A,
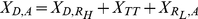
(22) as:
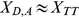
(23) and expand the reaction boundary of 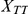 in Equation 20 to include the chemical potentials of the external ECs. Furthermore, one can obtain from Equation 21, the equilibrium constants for the exchange reactions 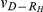 and 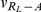:
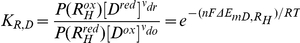


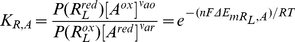
(24) which can be rewritten as the correlation between the internal concentration to the external concentrations:
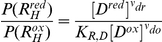


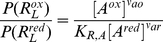
(25)


Using the algebraic constraints in Equation 13 and Equation 14, the relationships between the probabilities of each internal state and the concentrations of the external reactants are expressed as:
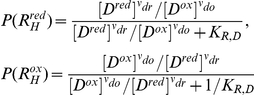


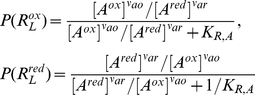
(26)


Here shorthand notations for the ratios of the redox states are introduced:
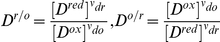


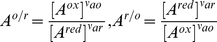
(27)


Substituting the relationships in Equation 26 and 27 for all the internal probability values in Equation 19 and Equation 20 allows the reaction flux to be expressed in terms of the external reactant species:
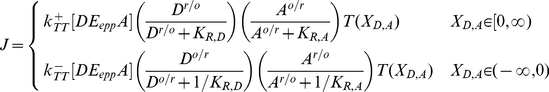
(28) where
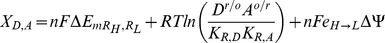
(29)


Noting the exponential form of 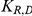 and 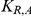 in Equation 24 and combining them with the midpoint potential 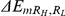 gives
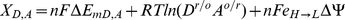
(30) which is nearly the same as the definition of 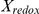 in Equation 5 and 6 (identical if 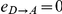 or if the electron is assumed to traverse the membrane between 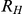 and 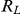 only).

Here the redox state function is introduced as:
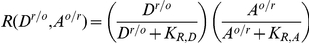


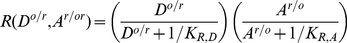
(31)which encapsulates the modulation on 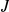 by the redox state ratios, and simplifies the expression of 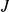 to:
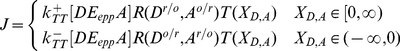
(32)


#### Saturation Function 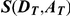


Equation 32 describes the flux as a forward rate coefficient 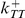 that is scaled by the concentration of the ternary complex 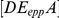, and modulated by both a thermodynamic force function 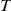 and a redox state function 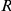, both of which change with respect to changes in the donor and acceptor redox state ratios. However, it is important to note that the two functions describe only the redox electron transfer processes of the 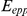 complex, but not the binding kinetics of the complex with respect to the donor and acceptor ECs (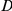 and 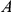). Indeed, binding kinetics is a central focus of several kinetics based models of ETC complexes, such as the Yugi and Tomita model [26], [27] or the more recent Chen and Beard model of complex I [44], because most enzyme regulation studies deal with the binding of substrates. However, the binding mechanism for each ETC complex may be unique and may also vary significantly across different mitochondrial systems, as suggested by the lack of consensus in the literature. Thus the strategy adopted here is to maintain the generality of the rate law so that it remains compatible with different commonly found mechanisms.

**Figure 6:**
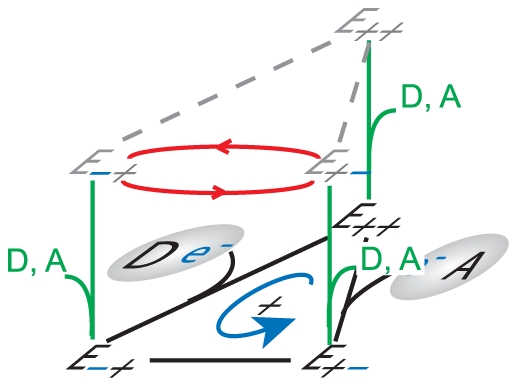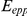 Reactant Binding Diagram. A simplified representation of how the binding of the EC reactants (green transition lines) separates the futile cycles (red transition lines) from the active redox cycles (original redox kinetic diagram).

At steady-state, the electron exchange reactions between the ECs and the 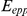 complexes are assumed to have achieved a rapid-equilibrium. Furthermore, since the binding of the EC reactants to form the ternary 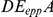 complexes precedes the exchange reactions, one can assume that the binding reactions must also be a part of the transient kinetics that takes place before the establishment of the steady-state. This enables us to superimpose the binding kinetics of EC reactants on Equation 32 by finding the fraction of the total 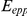 complex ensemble that forms 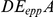. An extended kinetic diagram in Figure 6 illustrates how the binding of the ECs separates the 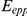 microstates from wasteful cicles and transitions to the active cycles that contribute to the reaction flux. The more the 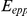 ensemble is “saturated” with the reactants, the higher the probability that the 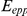 ensemble manifests itself in the 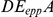 form. The extent of this saturation can be quantified by the ratio 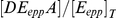, where
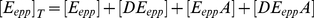
(33)


Using the nomenclature of Cleland [45], the binding mechanism for a typical two-substrate ETC redox reaction
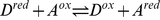
(34) can be classified by the reactant binding sequence (ordered sequential, random sequential, and ping-pong), and molecularity (uni, bi, ter, quad, etc) [46]. In an ordered sequential mechanism, substrates binding to and products release from the enzyme follow an exact order. In a random sequential mechanism, the order of binding between the two substrates or the order of release between the two products are random. In a ping-pong mechanism, one or more products must be released before all substrates can react. Since the ping-pong mechanism requires two separate catalytic steps, whereas a major assumption for the 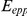 complex is the single rate-limiting catalytic step 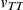, it is not incorporated at this time. The general scheme for reversable ordered sequential bi bi mechanism and reversable random sequential bi bi mechanism are
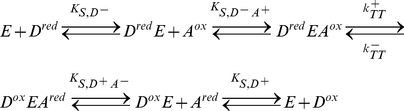
(35) and
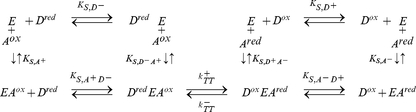
(36) respectively, where 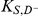, 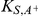, 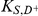, and 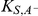 are the equilibrium dissociation constants for the binary 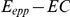 complexes, while 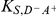, 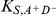, 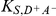, and 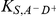 are the equilibrium dissociation constants for the ternary 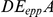 complex. Since the relative difference in the oxidized and reduced species of either the donor or the acceptor EC are already accounted for in the redox state function 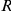, one can combine the contribution of both redox species of an EC reactant into one state variable through the constraints:
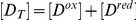


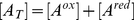
(37)


These constraints reduce the schemes in Equation 35 and 36 to pseudo-isomerization reactions
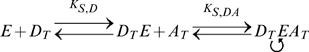
(38) and
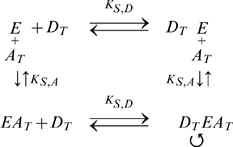
(39) whose solutions are identical to the solution for irreversible ordered sequential bi bi
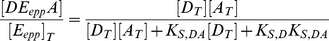
(40) and for irreversible random sequential bi bi
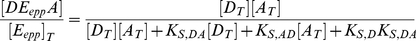
(41) as found in [45], [46].

In the situation where only one of the two substrate EC concentrations is varying while the other is held constant, one can essentially consider the effects of the individual variation separately as two separate and parallel reactions, each with a single substrate-enzyme binding step



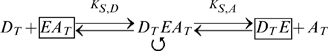
(42)


At steady-state, such independent binding reactions can always be expressed using the Michaelis-Menten-type kinetics [41]. The effects of the two independent reactions can be combined multiplicatively to give
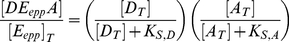
(43) where 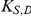 and 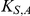 are the Michaelis-Menten-like saturation parameters that are characteristic of the enzyme complex. Equation 43 can be rearranged to give a comparable form to the sequential mechanisms:
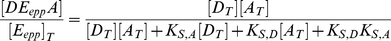
(44)Here a saturation function is introduced as:
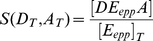
(45)which could be applied to any of the three variations shown in Equations 40, 41, and 44. Substitute 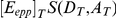 for 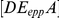 in Equation 32 and replace 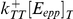 and 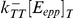 with the more familiar biochemical variable 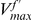 (apparent maximum forward velocity) and 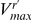 (apparent maximum reverse velocity), one obtains the expression
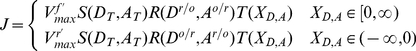
(46)which we label as the standard form of the chemiosmotic rate law. This standard form applies to the reference system which only contains the redox force of Equation 5. Adding other forces would transform the system and consequently change the form of the rate law. Combining the method in Equation 18 to set 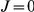, and the method in Equation 24 to set 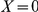, the equilibrium constant of the reaction in the reference system is found to be
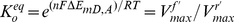
(47)which relates the ratio of the two maximum velocities to the midpoint redox potential of the reaction.

#### System Transformation

One important point in using Equation 46 is that although Equations 8 and 9 are equivalent over the entire domain of 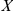, they are only equivalent if the equality in Equation 7 is not “perturbed”. If the composition of 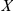 changes such that an additional force 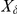 acts on the system, then the equilibrium point of the flux ratio ought to change with respect to the new force, and correspondingly the left hand side of Equation 7 ought to change in the same amount, giving the new relationship:
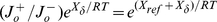
(48)where 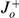 and 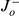 are the original forward and reverse fluxes with respect to a reference thermodynamic force 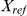. Since 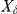 could separately affect 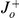 and 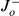, it creates a continuum of possibilities that could satisfy Equation 48, all of which can be encompassed by the introduction of a single dimensionless parameter 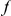:
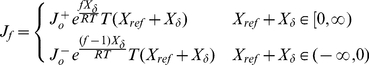
(49)where 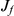 is the final reaction flux, and 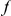 in 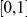 represents the fraction of influence 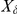 has on 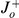 and 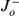. Note that if 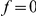, 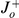 is unaltered while 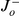 bares all the influence from 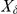; conversely, if 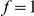, 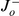 is unaltered while 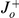 bares all the influence from 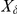. The exponential terms 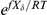 and 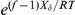 together represent the operations necessary to transform the original reaction flux 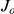 of the 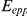 complex from a reference system to a new system with the added force. In general, for each additional force, an additional pair of exponential terms is applied to Equation 49. Thus, for a number 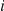 of additional forces, the 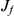 of the final system is expressed in terms of the 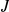 of the original system by:
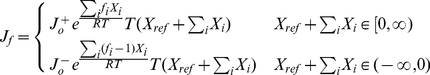
(50)


#### Free Energy Transduction and Enzyme Slippage (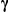)

The standard form of the chemiosmotic rate law in Equation 46 considers only the redox thermodynamic force 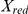 of the reference system. Such a system exists when proton gradient is not available either because the system cannot compartmentalize protons (i.e. a continuous membrane is not present to keep proton concentrations apart), or because the proton concentrations exactly balance across the membrane through clamping, both of which are observable and controllable in *in vitro* experiments. Thus, the reference system represents a fundamental basis from which the rate law for many other *in vitro* or *in vivo* systems can be derived through the transformation framework of Equation 50.

**Figure 7:**
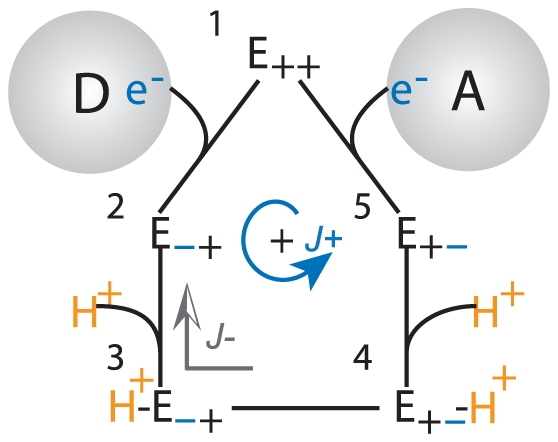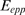 Free Energy Transduction Kinetic Diagram. The free-energy transduction processes in the 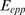 enzyme transition through a cyclic sequence of five states in both forward and reverse directions (indicated by the forward flux 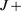 and reverse flux 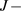), in which the enzyme: (**1**) starts in its free state 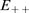; (**2**) binds with electrons 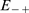; (**3**) binds to protons 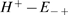; (**4**) couples the internal electron transfer with the change in conformation 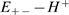; (**5**) loses protons 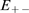; and finally loses an electron to return to the original free state 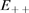 in the first state.

The incorporation of proton gradient can be viewed as such a transformation. The free energy transduction reaction in a chemiosmotic complex is driven by an overall thermodynamic force 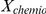 that is the sum of the two opposing forces:
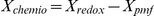


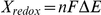


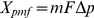
(51)where 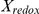 is the redox force defined in the reference system, 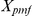 is the proton motive force (*pmf*), 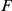 is the Faraday constant, and 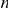 and 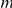 are the stoichiometric values for the number of electron transferred and net proton translocated respectively. In the *pmf*,
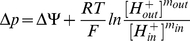
(52)is the energy necessary to pump one proton across the membrane with respect to the proton gradient and the membrane potential. Schematically, the addition of 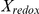 transforms the kinetic diagram of the redox reaction in Figure 4 to the diagram of the transduction reaction in Figure 7. Energy transduction occurs when the free energy of the electron carriers decreases by an amount 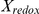, and from this, an amount 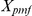 is used to increase the free-energy of the protons. At equilibrium, 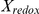 is in balance with 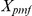, but an imbalance between the two would produces a net thermodynamic drive. In accordance with Equation 49, 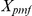 is a negative perturbation force 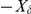 upon the reference system, and the additional parameter 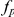 is used to determine the fraction of the effect of the the perturbation force that is distributed on the reference forward 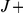 and reverse 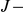 fluxes. The transformed expression of 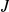 is then:
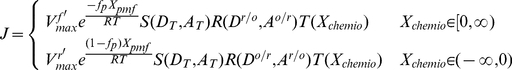
(53)


Since the equilibrium constant can be expressed as the ratio of the rate constants, the equilibrium constant of the transformed reaction with respect to the reference reaction is then
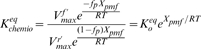
(54)


**Figure 8:**
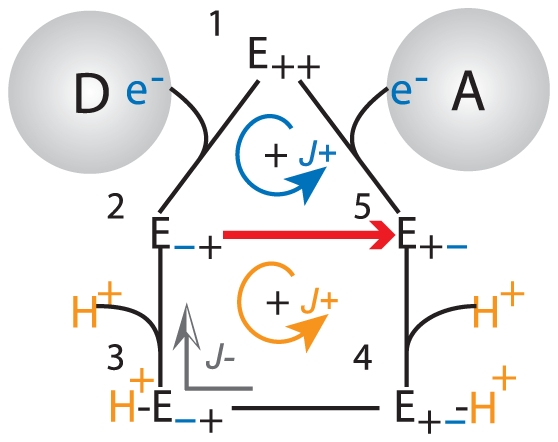
Electron Slippage. An alternate electron path between states (2) and (5) is introduced in the enzyme transition mechanism that short-circuits the normal cycle of the enzyme and uncouples the free energy transduction to drive the proton translocation.

In general, a transformation such as the addition of the 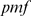 would shift the equilibrium constant of the original reaction (Equation 54); however, if the sum of perturbations in Equation 50 affects the forward and reverse rate constants in an equal but opposite manner, the original equilibrium will be preserved. Another possibility is if the perturbation force is a function of the original thermodynamic force, then the equilibrium concentrations of the reactants will not changed (albeit the equilibrium constant would be modified). One such perturbation is the “slippage” in the free energy transduction process. Energy transduction processes are prone to slippages in which efficiency can be affected by several factors [47] such as increased proton leakage or the loss of electrons to form ROS [6]. As a simple illustration, the efficiency of the flux-force relationship in Equation 7 can be compromised if a short circuit occurs in the cyclic states of the free energy transduction process (Figure 8). Alternate enzyme transition cycles could diverge from the normal transduction path, dissipating portions of the free-energy acquired from the high energy substrate without performing the transduction on the secondary substrate. This decrease in the available free-energy, expressed in terms of a smaller thermodynamic force, would lessen the magnitude of the net transduction flux according to Equation 11. To describe this lost of thermodynamic force 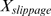 without explicitly expressing its content, a convenient 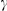 variable is introduced:
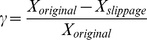
(55)such that 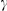 represents the percent of original thermodynamic force 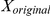 available after losing a fraction through the slippage transition path. 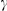 has the range between 0 to 1 as 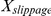 has a upper bound of 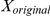. Setting 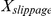 as the perturbation force in Equation 49 but expressing 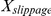 in terms of 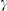 and 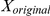 with Equation 55 gives the slippaged corrected expression of 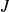 as
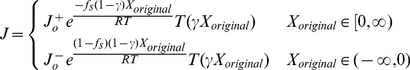
(56)


Note that because the transformed thermodynamic force is a fraction of 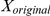 (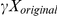), both of them would have the same equilibrium point at 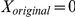. Thus, the perturbation by slippage does not change the equilibrium point in the reaction coordinate. Combining the result from Equations 53 and 56 into Equation 50, one obtains the general expression for a free energy transducting chemiosmotic rate law with slippage:
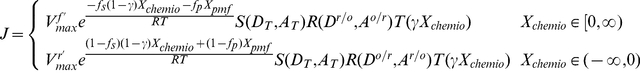
(57)


### Methods for Determining the Kinetic Parameter Values

**Figure 9:**
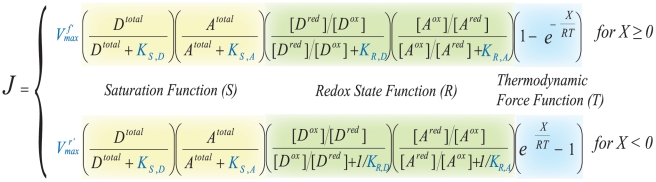
The Standard Form of Chemiosmotic Rate Law. The rate law equation consists of four components which contain six kinetic parameters that are experimentally determinable (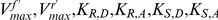 in blue).

The standard form of the chemiosmotic rate law, assuming a parallel binding mechanism (Equation 43), contains a total of six kinetic parameters: apparent maximum forward and reverse velocities 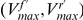, donor and acceptor reactant saturation constants 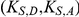, and donor and acceptor redox state constants 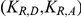 (Figure 9). Through the equilibrium constant in Equation 47, the six parameters can be reduced to five as either 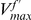 or 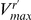 can be expressed in terms of the other. One of the main advantages of this rate law is that all five of its basis kinetic parameters can be fully determined through enzyme kinetic studies (e.g. the consensus protocols of respiratory chain spectrophotometric assays for clinical diagnosis http://lbbma.univ-angers.fr/lbbma.php?id=58). The principle technique for these assays involves the use of an ultraviolet/visible (UV/VIS) absorption spectrophotometer in which the time-course conversion of a redox substrate species to its product species by an ETC complex in a closed system (inside a curvette) is recorded to obtain the initial velocity (rate) of the reaction. Homogenated tissues and isolated mitochondria (where membranes are fragmented in both cases) are specifically used as the reference system since its *pmf* can be neglected due to the absence of an intact mitochondrial inner membrane. Thermodynamic constants from the literature and saturation reactant concentrations used in our experiments are listed in Table 2. For our studies, the protocol is extended to provide a complete time-series of the reaction until the substrate species is completely exhausted or the system reaches an equilibrium.

**Table 2:** Experimental Constants.

	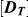 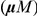	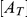 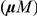	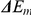 (mV)[2]
CI ( 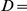 NAD, 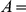 CoQ)	100	100	359.49
CIII ( 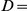 CoQ, 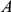 = cyctC)	100	50	168.58
CIV ( 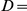 cyctC, 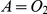 )	50	Bulk	318.55

In the following sections, procedures to determine the five kinetic parameters from the time-series data are described. With the exception of the kinetics for the final electron acceptor in complex IV, where both oxygen and water are reactants that are open to the bulk concentration, these procedures apply to all kinetic parameters in the ETC complexes. All of the fitting and subsequent simulations of the rate equation are performed using Mathematica 8′s *NonlinearModelFit* function, which produces least-squares fits that are defined to minimize the quantity 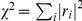, where the 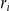 are residuals giving the difference between each original data point and its fitted value. The procedures and results are available in the form of Mathematica notebook files at http://www.igb.uci.edu/tools/sb/mitochondria-modeling.html.

#### Determining the Maximum Forward Velocity (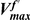) and the Saturation Parameters (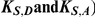


**Figure 10:**
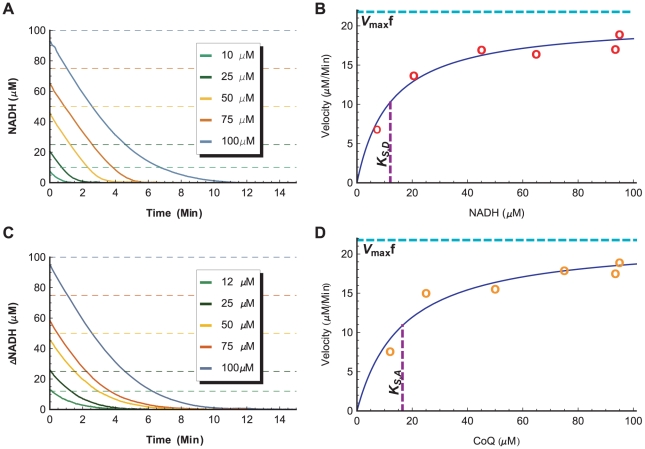
Determination of the Maximum Forward Velocity 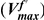 and the Saturation Parameters 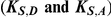. A. Complex I experimental time-series with varying starting 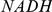 concentrations while 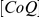 is held at 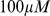. Dashed lines represent pipetted (targeted) NADH concentrations. B. The corresponding fit of the function 
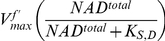
 to the initial velocity (rate of change of concentration) and the actual starting 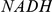 concentration of each time-series in the 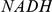 variation assay set plus an overlapping time-series from the 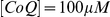 assay. C. Complex I experimental time-series with varying starting 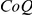 concentrations while 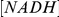 is held at 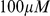. Due to poor separation between 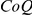 and 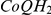 absorption frequency in spectrophotometry assays, time-series are obtained by following the relative changes in the 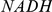 concentration 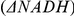 instead. D. The corresponding fit of the function 
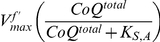
 to the initial velocity and the actual starting 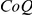 concentration of each time-series in the 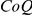 variation assay set plus an overlapping time-series from the 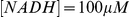 assay.

As indicated in the derivation of Equation 45, out of the three modulating function, the 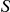 saturation function affects the reaction flux first. Thus its two saturation parameters, 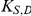 and 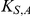, can be obtained from the instantaneous initial velocity of the reaction when there are no products so that functions 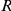 and 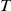 have negligible contributions. Furthermore, since it is assumed that the two binding reactions in 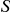 are independent to each other, 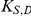 and 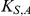 can each be determined separately by varying the starting concentration of the corresponding EC substrate while saturating the EC substrate of the other parameter to minimize its contribution. Consequently, for each complex parameter determination, there are necessary two sets of time-course enzyme kinetics assays: (1) the 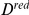 set with variations in the initial concentration of the reduced donor substrate specie; and (2) the 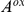 set with variations in the initial concentration of oxidized acceptor substrate specie. To illustrate, Figure 10A shows the time-series of complex I with variations in the initial concentration of 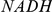, while Figure 10C shows the time-series of complex I with variations in the initial concentration of 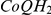.

For each assay set, an initial velocity is approximated for each of the initial concentrations by calculating the change in concentration over a starting time period (dependent on the amount of enzymes used). The initial velocity-concentration pairs are then used as data points for calculating the residuals in the least-square fitting of the corresponding Michaelis-Menten-like factor in Equation 43 to obtain the associated saturation parameter (Figure 10B and 10D). A final value of 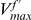 is obtained by averaging the fitted initial velocity value from both 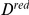 and 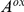 variation sets.

#### Determining the Redox State Parameters (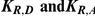)

Beyond the transient period of the instantaneous initial velocity, both 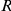 (Equation 31) and 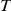 (Equation 10) contribute significantly in the modulation of the reaction flux. The 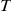 function can be fully described by the time-dependent variables 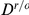 and 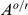 and the thermodynamic constant 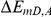 (Equation 30 with 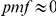), leaving only the 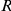 function to be determined. 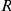 imposes on the flux, a hyperbolic dependence on the ratios 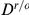 and 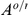 through the redox state parameters 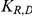 and 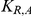, respectively. Both ratios change continuously as the reaction progresses, and their rate of change are related through the stoichiometric coefficients of their constituent species in the overall reaction (Equation 1 and 2); therefore, the effects of 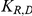 and 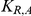 are not separable in the reaction, and must be considered together.

**Figure 11:**
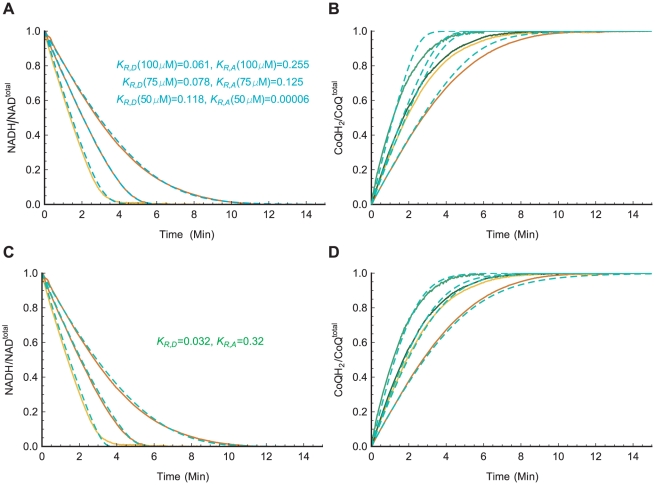
Determination of the Redox State Parameters (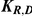 and 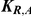). Complex I 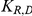 and 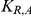 parameters are estimated simultaneously through the least-square fitting of the Chemiosmotic rate law to the experimental time-series from the 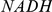 variation assays. The fitted parameters are then used to simulate and compare to the experimental time-series from the 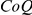 variation assays. To provide a common range of value to compare the time-series, all experimental and simulation data are normalized by the respective total reactant concentration. A. Parameter fitting using individual time-series of 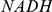 variation assays (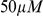, 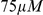, and 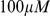 from left to right). Although the individual simulation output (in cyan) matches closely to the experimental data (residual values: 0.1, 0.01, and 0.05 respectively), the corresponding parameter values varies significantly (values shown in the Panel). B. Simulation results from the 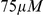 time-series fitted parameters in Panel A reveal a poor match (total residual value: 10.4) to the time-series from the 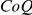 variation assays (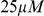, 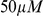, 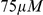, and 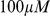 from left to right). C. Parameter fitting using all three time-series from Panel A. Here the simulation outputs for the three time-series (represented by the green dashed lines and based on the single set of 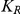 parameter values shown in the Panel) show a looser fit to the experimental data (residual values: 0.2, 0.1, and 0.15 respectively). D. However, the estimated parameter values from the combined fitting produce a much better match (total residual value: 0.58) to the time-series from 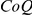 variation assays.

Given all other parameter values are fixed or determined, the values for 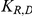 and 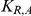 can be determined by fitting the simulation output from Equation 46 to the time-series from the 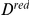 or 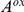 variation assay sets. Although each time-series from the two sets of assays differs in its total reactant concentrations, the values of 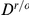 and 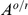 only depend on the concentration ratio of the respective redox-pairs at the specified time. Thus, ideally, every one of the time-series can supply the full value range of the ratios to obtain an estimate for 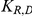 and 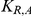. However, because 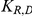 and 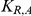 must be determined simultaneously, and the factors containing them in the 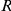 function are symmetrical, the estimated parameter values from fitting a single time-series are not unique and might not provide the optimal representation of the complex reaction over various conditions. This is demonstrated in Figure 11A and 11B, where the best fit parameter values estimated individually from the time-series 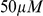, 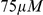, and 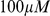 of the 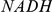 assays are shown to have a large variation in the parameter values, and the subsequent simulations of the rate law based on one of the three estimates are shown to have a poor agreement with the time-series from the 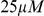, 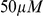, 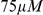, and 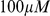 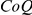 assays (due to the level of measurement noise in the time-series, 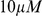 and 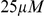 assays for 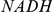 and 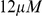 assay for 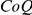 are not included in this analysis). To obtain a better estimate of the parameters, multiple time-series are used simultaneously instead in a combined least-square fitting across various initial concentrations. Even though individually the error value may increase between the simulation time-series and the experimental time-series used for the fitting, the estimated 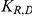 and 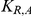 parameter values from the combined fitting produce simulations that agree with the complex's characteristics across a much greater range of conditions. This is shown in Figure 11C and 11D, where the same time-series from Figure 11A are simultaneously used in a combined fitting of the parameters, and the subsequent simulations of the rate law based on this combined fitting are then shown to give a much better agreement with the time-series from the 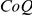 assays.

Note that the ratios 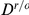 and 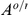 would approach infinity when the divisor concentrations (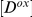 and 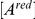) approach zero (refer to Equation 27). Thus to avoid numerical errors in the simulation, a small amount of 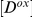 and 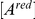 are assumed to have been created during the transient period before reaching the steady-state.

## Results and Discussion

### Comparison with Existing ETC Energy Transduction Equations

In Table 3 the new chemiosmotic rate law is compared to the ETC rate equations from both the Korzeniewski and the Beard OXPHOS models using complex I as the example. Each of the three rate equations is derived using a thermodynamics approach with the same formulation for the thermodynamic force (Equation 51) associated with the overall reaction of a given complex (Equation 1). However, in spite of their common origins, the three approaches differ in the level of detail at which the complex is modeled, which in turn results in functional differences.

**Table 3:** Comparison of Rate Equations for Complex I.

	Equation
Korzeniewski	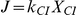
Beard	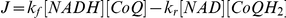 , 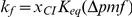 , 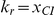 , 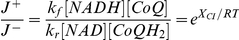
Chemiosmotic	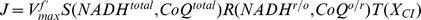 , 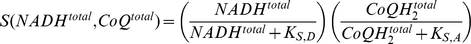 , 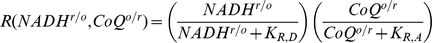 , 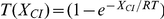
Common	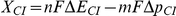 , 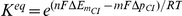

Korzeniewski's rate equation follows Onsager's linear force-to-flux relationship, based on the local equilibrium approximation where thermodynamic forces vary slowly [48]. In contrast, Beard's rate equation and the chemiosmotic rate law work with non-linear steady-states that are not bound to the same restrictions. In fact, the derivation of Beard's equation follows the same thermodynamic force-to-flux relationships up to Equations 7 and 8, but diverges in the representation of the forward and reverse fluxes in Equation 4, where it simply assumes a mass-action reaction. By modeling the overall reaction as an elementary mass-action reaction, Beard's equation does not account for the kinetics within the complex. Its single kinetic parameter, denoted the activity parameter 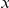, is a scaling parameter that serves to adjust the magnitude of the net flux (similar to 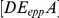 in Equation 32), but does not capture the maximum turnover rate of the enzyme or its affinity towards substrates. In contrast, the 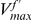 and 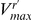 of the chemiosmotic rate law represent the apparent maximum velocities of the reaction, which are modulated by both thermodynamic and kinetic factors through the three bounded functions in Equation 46 (Figure 9).

**Figure 12:**
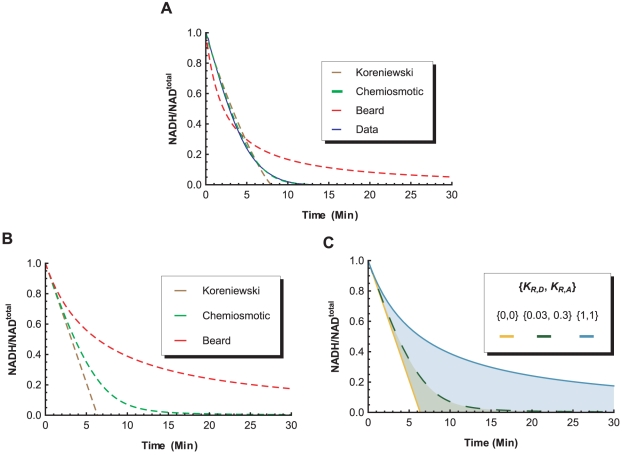
Comparison of Rate Equations. **A.** Least-square fitting of Koreniewski's formulation, Beard's formulation, and the Chemiosmotic rate law over the entire range of an experimental time-series from complex I (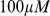 case). **B.** Least-square fitting of the three rate equations to the initial velocity of the experimental time-series. **C.** The output of the Chemiosmotic rate equation gives approximately the same results as the Beard's equation when 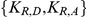  =  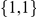, and approaches the output of the Koreniewski's equation when 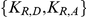  =  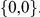

Least-square fitting of the three rate equations over the entire range of the experimental time-series data from an isolated complex I kinetics assay allow a comparison of how well the equations can reproduce the original time-series through their respective parameters. Figure 12A shows that the chemiosmotic rate law gives a better approximation of the experimental data compared to Korzeniewski's linear function, or Beard's mass-action based function. However, a more accurate comparison of the principles guiding the three rate equations is given by fitting them to the initial velocity of the time-series data, where the three rate equations should theoretically converge. Figure 12B shows that the output of the chemiosmotic rate equation, as well as the original experimental data, fall between the other two approaches. When the comparison is extended to all possible values of 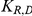 and 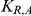 in the chemiosmotic equation (Figure 12C), one can note that the output of the chemiosmotic equation becomes equivalent to the output of Beard's equation when both 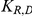 and 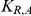 are equal to one, while it approaches the output of Korzeniewski's equation when both 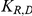 and 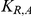 approach zero.

This can be explained by noting that when both 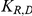 and 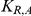 are equal to one in Equation 25, there is a direct correlation between the external concentrations and the internal probabilities, and Equation 19 becomes essentially the same as the Beard's first-order mass-action reaction representation of complex I. On the other hand, when both 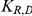 and 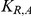 approach zero, all probabilities in Equation 26 approach one. This results in a zero-order reaction that is insensitive to changes in the external concentrations, which is similar to the simulation output of Korzeniewski's linear flux equation.

An important result emerging from these comparisons is that the new chemiosmotic rate law represents a more general formulation of the kinetic and thermodynamic behaviors for a chemiosmotic ETC complex; one which encompasses both Korzeniewski's and Beard's formulations, and can smoothly interpolate between them to cover a spectrum of biochemical behaviors. Therefore, for a given overall reaction and a thermodynamic force definition, the chemiosmotic rate law can replace the existing ETC rate equations in the OXPHOS models of Korzeniewski and Beard to incorporate biochemically relevant kinetic parameters which allows a more accurate specification of an ETC complex.

### Kinetic Parameter Values and Sensitivity Analysis

This section covers the analysis of the kinetic parameter values and their sensitivity in the chemiosmotic rate law applied to each of the ETC complexes in isolation. A summary of the experimental values obtained for all the kinetic parameters of complex I, III, and IV are given in Table 4, and the corresponding sensitivity in Table 5. In addition, the sensitivity of the slippage parameter 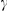 with equal perturbations on the forward and reverse rate constants (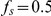) is also included for comparison. The sensitivity of all parameters are determined by calculating the magnitude of variations needed to change the flux of an isolated complex 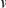 by 1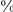. Thus, the lower the values in Table 5, the more sensitive the flux is to changes in the corresponding parameter.

**Table 4:** Experimentally Determined Parameter Values.

	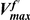 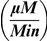	specific 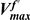 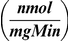	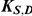 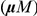	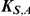 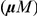	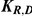	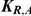
CI (20 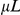 mito)	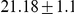	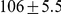	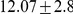	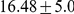	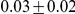	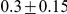
CIII (1 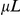 mito)	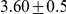	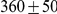	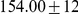	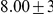	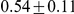	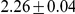
CIV (3 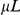 mito)	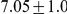	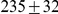	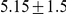	1.0 [29]	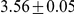	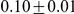
	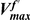 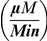	specific 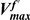 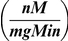	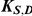 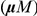	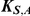 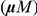	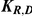	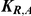
CI (20 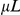 mito)	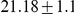	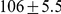	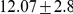	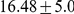	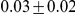	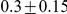
CIII (1 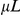 mito)	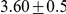	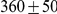	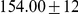	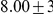	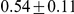	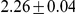
CIV (3 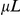 mito)	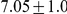	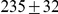	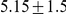	1.0 [29]	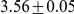	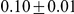

**Table 5:** Parameter Variation Resulting in 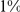 Change in Flux 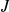.

	Complex I	Complex III	Complex IV
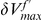	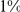	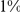	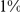
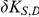	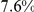	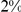	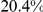
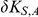	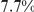	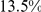	N/A
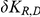	6,000X	5,232X	220X
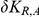	207X	255X	7,625X
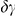	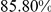	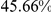	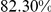

**Figure 13:**
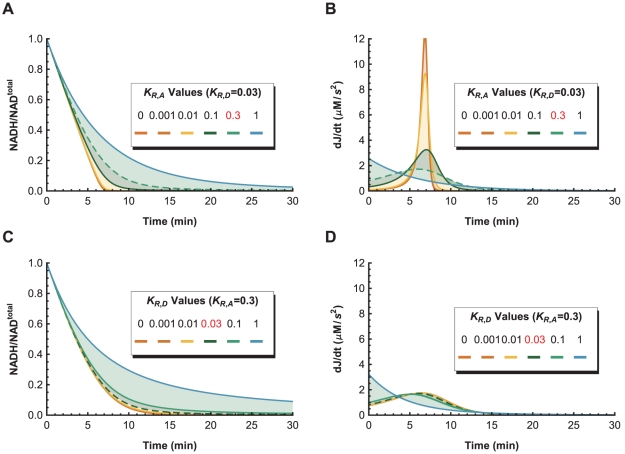
Sensitivity of 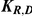 and 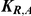 Values. **A.** The concentration time-series with respect to changes in 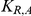 while 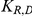 is held at 0.03 show that variations in 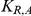 produce a subset of the time-series in Figure 12C close to the zero-order linear approximation. **B.** The curvature (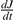) time-series obtained from the second-derivative of the concentration time-series in Panel A show that the closer the concentration time-series is to the linear approximation, the larger the changes to the curvature time-series. **C.** The concentration time-series with respect to changes in 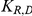 while 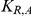 is held at 0.3 show that variations in 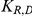 produce a subset of the time-series close to the first-order mass action approximation. **D.** The curvature time-series obtained from the second-derivative of the concentration time-series in Panel C show that the closer the concentration time-series is to the mass-action approximation, the smaller the changes to the curvature time-series, and also the lower the peak time of the curvature.

In summary, the sensitivity of 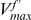 provides the least amount of information about a specific complex since it is constant across different complexes regardless of its parameter value or concentrations of reaction species. This is due to the fact that 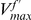 is independent from all other parameter and variable values in Equation 46. Variations 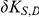 and 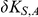 depend only on their respective parameters 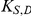 and 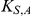, and total substrate concentrations 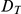 and 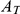. The smaller the value of 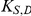 or 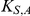 is compared to its respective total substrate concentration, the larger the parameter variation is required to change the flux, and therefore the less sensitive the parameter is. Compared to the other parameters in Table 5, where only small variations 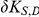, 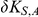, and 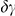 are needed to affect the flux, multiple-fold changes of 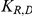 and 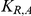 are necessary in order to affect the flux. These large values of 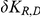 and 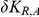 suggest that the flux is not very sensitive to changes in 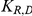 or 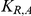. However, as shown previously in Figure 12C, small changes in the values of 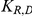 and 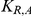 can give rise to significant changes in the curvature of the simulated reaction time-series. This is due to the fact that 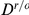 and 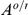 are time-dependent ratios of the species concentrations, which tend to infinity when there are only substrate species present, or become zero when there are only product species left. Thus, in the beginning of a time-series, when the values of 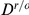 and 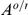 are large, 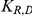 and 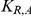 are small in comparison, and their variations have negligible effect on the flux. However, as 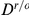 and 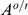 get smaller and closer to the values of 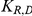 and 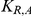 over time, their effect on the flux would become much more significant. In addition, the larger the value of the varying parameter, the earlier the reaction flux is affected by the concentration of the substrate (Figure 13). On the other hand, the smaller the value of the varying parameter, the longer the reaction maintains the same flux, resulting in a sharper change in the flux at the end of the time-series, when the reaction runs out of its reactant(s).

Whereas a 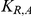 or 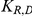 value of zero makes the reaction independent from the respective reactant concentration and a value of one makes the reaction dependent on the reactant concentration in a first-order mass-action fashion, a value larger than one suggests an even higher dependence on the reactant concentration than first-order. In addition, from Equation 24, when either 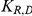 or 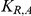 is larger than one, the redox potential of the respective boundary electron transfer reaction is necessarily negative, indicating an energetically unfavorable reaction. These derived relationships are important for the interpretation of the experimental values of 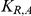 and 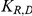 in Table 4. For complex I, both 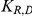 and 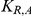 are lower than one. In contrast, for both complex III and complex IV, the cyctochrome c associated 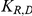 or 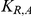 value is larger than one, suggesting a higher-order dependence on the concentration of cytochrome c consistently with the fact that two cyctochrome c are required for the corresponding reaction. Thus, even though complex III and IV are not very sensitive to the saturation concentration of cytochrome c, the redox state ratio of cytochrome c has a profound effect on the flux through the redox state function 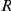.

Lastly, from Equation 55, it is clear that the effect of 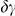 depends largely on the redox potential of the system (Table 2).

### Network Sensitivity Analysis

In this section, we conduct a more global sensitivity and perturbation analysis with the same experimentally observable *in vitro* homogenate environment, but with all the ETC complexes functioning in tandem. The simple pathway model of the ETC used for this analysis consists of interactions between a driving dehydrogenase reaction, the three main electron transfer complexes (complex I, III, and IV), and the three electron carrier redox pairs (Figure 1A). Each complex is modeled using the chemiosmotic rate law applied with the parameter values derived from our experiments (Table 4), together with thermodynamic constants from the literature and saturation reactant concentrations used in our experiments (Table 2).

To analyze the sensitivity of a network, the most commonly used tool is the flux control coefficient (FCC) [49], which represents the relative change in the global steady state flux 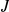 resulting from an infinitesimal change in a property of an individual complex 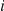, divided by the relative change of that complex's activity 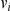 from the same infinitesimal change, and normalized by the corresponding steady state flux and complex activity. The complex with the largest FCC exerts the largest control on the flux at a particular steady state, as an increase in the activity of this complex would result in the largest overall flux increase. For the present study, an expanded definition of FCC is used such that the properties of interest are the individual parameters 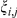 of the 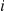
th complex:
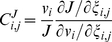
(58)


This expanded definition expresses quantitatively the effect that small variations in the parameter 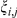 have on the flux of the system 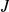, if the effect of 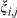 on the local complex activity 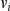 is known. The FCCs are calculated for the simple ETC pathway by taking the same parameter variation as in Table 5 to find the corresponding changes in 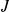 numerically, and then dividing the result by 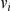. The results are summarized in Table 6, which shows that regardless of which parameter is varied, the control coefficients remain consistent for the same complex. This indicates that for the same small variation in 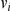, the parameters have the same effect on 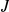 without significantly affecting the steady state concentrations.

**Table 6:** Flux Control Coefficients for the Parameters of ETC Complexes.

	Complex I	Complex III	Complex IV	Sum
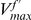	0.0283	0.4751	0.5035	1.0069
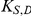	0.0241	0.4669	0.5087	0.9997
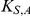	0.0241	0.4660	0.5111	1.0014
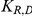	0.0205	0.4693	0.5090	0.9988
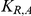	0.0241	0.4683	0.5126	1.0050
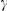	0.0241	0.4672	0.5096	1.0009

Although FCCs can show the relative control of the individual components in a pathway network, they have little predictive value as each FCC is computed for a single steady-state. As the state of the system changes, the values of the FCCs, may change as well. To overcome the single steady-state limitation of the FCC, we next apply threshold curve analysis [13] to study the effects of different parameter perturbations on our simplified ETC network across a continuum of steady-states. Compared to the FCC, a threshold curve is not limited to just infinitesimal changes in a parameter 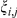, but gives a complete range of variations that result in 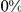 to 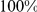 decrease in both 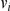 and 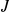. The percent decreases in 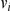 and 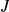 are plotted versus each other, and the relationship curve between them gives not only a measure of the global flux control by an individual enzyme complex, but possibly also a threshold value for 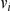 beyond which the level of 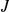 would be significantly reduced.

**Figure 14:**
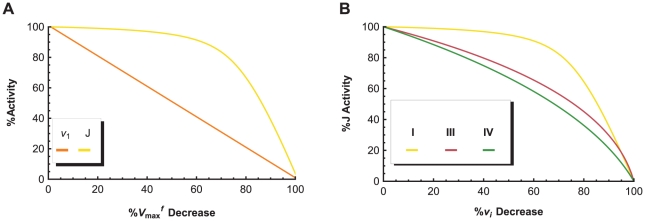
Threshold Curves and Threshold Profile. Threshold curves relate the changes in an individual complex's activity (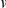) with the changes in the global steady-state flux 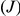 of the network, through the changes in a parameter of the rate equation. **A.** A change in the value of complex I's 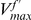 parameter has a direct corresponding change in the complex I's isolated activity (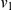), but a more buffered effect on 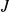, where a threshold value can be identified. **B.** The two data series in Panel A are plotted against each other to represent the threshold curve of complex I. Together with the threshold curves of complex III and IV, this combination plot represents the threshold profile of the network/system.

An example is shown in Figure 14A, where variations in the 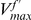 parameter of complex I have a direct one-to-one effect on the complex I activity 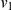, but a delayed effect on 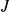. The two curves are combined to form the complex I threshold curve in Figure 14B, where it is paired with the threshold curves of complex III and IV to produce the specific threshold profile of the system. The threshold effects shown in these curves can be attributed to a combination of: (1) an excess of complex activity due to an excess of complex available in the system, which tends to produce a curve with a plateau phase, followed by a sharp decline in 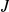; and (2) the buffering of individual complex activity perturbations by the metabolic network (kinetic properties of the enzymes, structure of the pathway network, concentrations of substrates, etc.), which is responsible for the smoothness of the curves [50]. As such, the clear threshold value of complex I, and the smooth threshold curves of complex III and IV indicate that complex I is in excess relative to complex III and IV, which affect 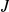 in a more gradual and controlling manner. Thus, the threshold profile gives the same conclusion as the FCC analysis, but offers a more complete view of a network's interactivity across the entire normalized range of 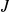 steady-states.

**Figure 15:**
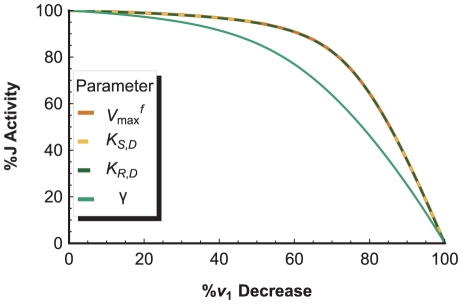
Threshold Curves of Various Parameters. This comparison of different complex I threshold curves shows that variations in both the saturation parameters (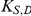 or 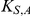) and the redox state parameters (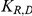 or 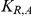) produce the exact same threshold curve as the 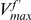 parameter, but the 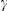 parameter produces a different curve due to the change in the thermodynamic property of the complex.

The same threshold profile can be observed irrespective of which kinetic parameter is used to establish the relationship between that of 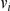 and 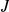, so long as the two are always compared at the same steady-state. Figure 15 shows that this is true for the complex I threshold curve with respect to modulations in the 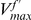 parameter, the saturation parameters (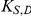 or 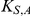), and the redox state parameters (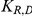 or 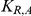). However, in the case of the 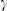 parameter, the threshold is markedly changed because the thermodynamic property of the complex, and the structure of the network are changed with the introduction of an alternative pathway for the electron in the complex.

**Figure 16:**
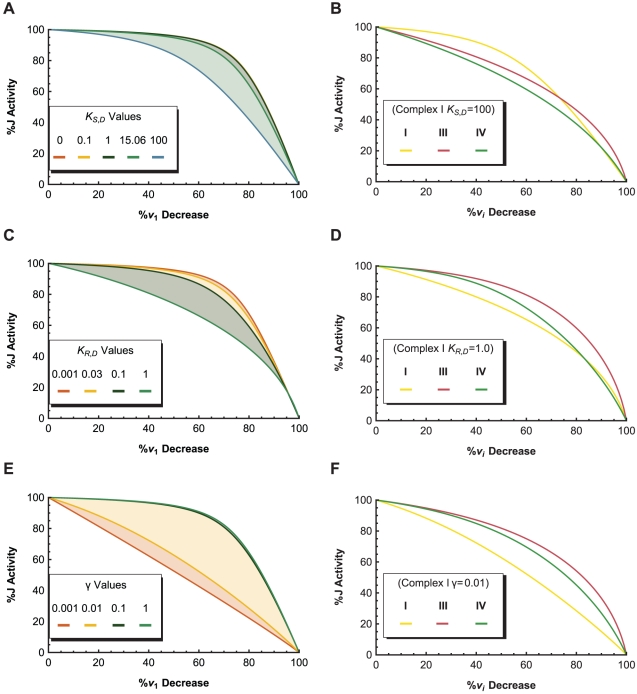
Parameter Perturbations and the Threshold Curve. Each parameter in the rate law perturbs the complex I threshold curve and threshold profile in a different way. **A.** The saturation parameters (represented by 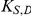) do not alter the metabolic network significantly, which allows the threshold curve to maintain its shape and a threshold value. **B.** At 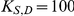, there are no perceivable effect on the threshold curves of the other two complexes. **C.** The redox state parameters (represented by 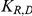) have a significant effect on the metabolic network, and thus the shape of the threshold curve as it changes 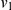
's order of dependency on the reactant concentrations. **D.** At 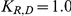, the entire threshold profile is greatly affected by the changes in the metabolic network. **E.** The 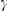 parameter has no effect on the threshold curve until its value drops below 0.1. After which, the threshold curve quickly approaches that of a straight line. **F.** At 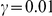, 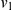 becomes the sole rate-limiting reaction.

The threshold profile of the system is consistent for all kinetic parameters because the threshold curve relates the relative changes instead of the absolute changes in 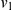 and 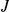. Thus, threshold curves based on different kinetic parameters cannot show how each parameter may alter the network. Instead, we look at how the threshold profile of the system is changed by a single value perturbation in a specific parameter. This is demonstrated in Figure 16, which compares how perturbations in the saturation parameters, redox state parameters, and the 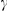 parameter affect the complex I threshold curve and the threshold profile of the system. A visual metrics of comparing the threshold between the threshold curves is presented as the area between two consecutive curves, which represents the difference in the area under the two curves (color coded also by the difference in the color index of the two curves). The quantitative measure of each threshold curve in threshold profile is presented in Table 7 as the fraction of the total square plot area covered under the curve. From Figure 16A, increases in the value of a saturation parameter (represented by 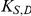) shifts the threshold value of the curve to the left but the plateau phase is maintained for a large range of parameter values. The perturbed threshold profile in Figure 16B shows that even at a value of 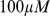, the effect of a saturation parameter is localized to the threshold of its own complex and has no significant effect on the threshold of the other two complexes. In contrast, Figure 16C shows that increases in the value of a redox state parameter (represented by 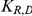) has a significant effect on the shape and smoothness of the threshold curve as it changes 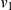
's order of dependency on the reactant concentrations, indicative of a large perturbation effect on the metabolic network. At 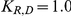, the perturbed threshold profile in Figure 16D shows that this perturbation in the metabolic network is propagated to complex III and IV, and affects their threshold curves greatly. The 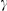 parameter has essentially no effect on the threshold curve in Figure 16E until its value drops below 0.1. This suggests that the thermodynamic force of complex I, in the absence of the *pmf*, has an excess in the redox potential, and that only ten percent of the redox potential is necessary to drive the redox reaction of complex I. After dropping below 0.1, the threshold curve quickly approaches that of a straight line, showing direct effect on the flux. The corresponding threshold profile in Figure 16D shows that, at 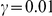, 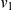 becomes so rate-limiting that 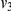 and 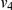 of complex III and IV are always in excess in relation to 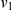.

**Table 7:** Measure of Threshold in Threshold Profile of Figure 16 (Fraction of the Total Area Under the Curve).

	Complex I	Complex III	Complex IV
Figure 16B ( 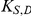 )	0.72	0.65	0.55
Figure 16D ( 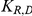 )	0.64	0.80	0.72
Figure 16F ( 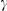 )	0.55	0.78	0.70

### Conclusion

In short, we have developed a framework for modeling mitochondria bioenergetics using a new chemosmotic rate law to represent each ETC complex in the OXPHOS pathway. This modular framework subsumes and generalizes several previous approaches and relies on a small set of biochemically relevant parameters. We have conducted enzymatic assays to derive those kinetic parameters for complex I, III, and IV, and validated the predictions of the model against experimental concentration time-series. These results, together with detailed sensitivity analyses, show that the parameters, originally derived from a simple reference system, provide a good breadth of feasible physiological responses of the complexes. In particular, threshold curves relating the flux of one complex to the global flux of the system show how each parameter in each complex can have a differential effect on the threshold curve of the corresponding complex, as well as on the overall threshold profile of the system (Figure 14B and 16). The flexibility and accuracy of the model, coupled with the diverse range of behaviors it is capable of generating through transformation of the system, suggest that in the future this approach could enable the comparative modeling and analysis of mitochondria from different systems and pathological states.

## References

[1] Mitchell P (1969). Chemiosmotic Coupling and Energy Transduction.. Theor Exp Biophys.

[2] Nicholls D, Ferguson S (2002). Bioenergetics 3..

[3] Demin OV, Kholodenko BN, Skulachev VP (1998). A model of o 2-generation in the complex III of the electron transport chain.. Molecular and cellular biochemistry.

[4] Kushnareva Y, Murphy AN, Andreyev A (2002). Complex i-mediated reactive oxygen species generation: modulation by cytochrome c and NAD (P)+ oxidation-reduction state.. Biochemical Journal.

[5] Brand M (2005). The efficiency and plasticity of mitochondrial energy transduction.. Biochemical Society Transactions.

[6] Loeb L, Wallace D, Martin G (2005). The mitochondrial theory of aging and its relationship to reactive oxygen species damage and somatic mtDNA mutations.. Proceedings of the National Academy of Sciences.

[7] Brandon M, Baldi P, Wallace DC (2006). Mitochondrial mutations in cancer.. Oncogene.

[8] Ruiz-Pesini E, Lott MT, Procaccio V, Poole J, Brandon MC (2007). An enhanced MITOMAP with a global mtDNA mutational philogeny.. Nucleic Acids Research.

[9] Brandon M, Baldi P, Wallace D (2009). MITOMASTER: A bioinformatics tool for the analysis of mitochondrial DNA sequences.. Human Mutation, Database Issue.

[10] Rocher C, Taanman J, Pierron D, Faustin B, Benard G (2008). Inuence of mitochondrial DNA level on cellular energy metabolism: implications for mitochondrial diseases.. Journal of Bioenergetics and Biomembranes.

[11] Boekema E, Braun H (2007). Supramolecular structure of the mitochondrial oxidative phosphory-lation system.. Journal of Biological Chemistry.

[12] Benard G, Bellance N, James D, Parrone P, Fernandez H (2007). Mitochondrial bioenergetics and structural network organization.. Journal of Cell Science.

[13] Letellier T, Heinrich R, Malgat M, Mazat J (1994). The kinetic basis of threshold effects observed in mitochondrial diseases: a systemic approach.. Biochemical Journal.

[14] Rossignol R, Faustin B, Rocher C, Malgat M, Mazat J (2003). Mitochondrial threshold effects.. Biochem J.

[15] Benard G, Faustin B, Passerieux E, Galinier A, Rocher C (2006). Physiological diversity of mitochondrial oxidative phosphorylation.. American Journal of Physiology- Cell Physiology.

[16] Letellier T, Malgat M, Rossignol R, Mazat J (1998). Metabolic control, analysis and mitochondrialpathologies.. Molecular and cellular biochemistry.

[17] Yao J, Irwin RW, Zhao L, Nilsen J, Hamilton RT (2009). Mitochondrial bioenergetic deficit precedes alzheimer's pathology in female mouse model of alzheimer's disease.. Proceedings of the National Academy of Sciences.

[18] Vo T, Palsson B (2007). Building the power house: recent advances in mitochondrial studies through proteomics and systems biology.. American Journal of Physiology- Cell Physiology.

[19] Zhou L, Salem J, Saidel G, Stanley W, Cabrera M (2005). Mechanistic model of cardiac energy metabolism predicts localization of glycolysis to cytosolic subdomain during ischemia.. American Journal of Physiology-Heart and Circulatory Physiology.

[20] Lales C, Parisey N, Mazat J, Beurton-Aimar M (2009). Simulation of mitochondrial metabolism using multi-agents system..

[21] Plank G, Zhou L, Greenstein J, Cortassa S, Winslow R (2008). From mitochondrial ion channels to arrhythmias in the heart: computational techniques to bridge the spatio-temporal scales.. Philosophical Transactions of the Royal Society A: Mathematical, Physical and Engineering Sciences.

[22] Cortassa S, O′Rourke B, Winslow R, Aon M (2009). Control and regulation of mitochondrial energetics in an integrated model of cardiomyocyte function.. Biophysical journal.

[23] Nguyen M, Dudycha S, Jafri M (2007). Effect of Ca2+ on cardiac mitochondrial energy production is modulated by Na+ and H+ dynamics.. American Journal of Physiology-Cell Physiology.

[24] Jin Q, Bethke C (2002). Kinetics of Electron Transfer through the Respiratory Chain.. Biophysical Journal.

[25] Jin Q, Bethke C (2003). A New Rate Law Describing Microbial Respiration.. Applied and Environmental Microbiology.

[26] Yugi K, Tomita M (2000). Quantitative modeling of mitochondrial energy metabolism using ECELL simulation environment..

[27] Yugi K, Tomita M (2004). A general computational model of mitochondrial metabolism in a whole organelle scale.. Bioinformatics.

[28] Korzeniewski B, Zoladz J (2001). A model of oxidative phosphorylation in mammalian skeletal muscle.. Biophysical Chemistry.

[29] Beard D (2005). A biophysical model of the mitochondrial respiratory system and oxidative phos-phorylation.. PLoS Comput Biol.

[30] Wu F, Jeneson JAL, Beard DA (2006). Oxidative ATP synthesis in skeletal muscle is controlled by substrate feedback.. AJP: Cell Physiology.

[31] Wu F, Yang F, Vinnakota KC, Beard DA (2007). Computer modeling of mitochondrial tricarboxylic acid cycle, oxidative phosphorylation, metabolite transport, and electrophysiology.. Journal of Biological Chemistry.

[32] Beard DA, Vinnakota KC, Wu F (2008). Detailed enzyme kinetics in terms of biochemical species: study of citrate synthase.. PLoS One.

[33] Beard DA (2006). Modeling of oxygen transport and cellular energetics explains observations on in vivo cardiac energy metabolism.. PLoS Comput Biol.

[34] Korzeniewski B, Noma A, Matsuoka S (2005). Regulation of oxidative phosphorylation in intact mammalian heart in vivo.. Biophysical chemistry.

[35] Guillaud F, Hannaert P (2008). Dynamic simulation of mitochondrial respiration and oxidative phosphorylation: Comparison with experimental results.. Acta Biotheoretica.

[36] Bazil JN, Buzzard GT, Rundell AE (2010). Modeling mitochondrial bioenergetics with integrated volume dynamics.. PLoS Comput Biol.

[37] Modre-Osprian R, Osprian I, Tilg B, Schreier G, Weinberger KM (2009). Dynamic simulations on the mitochondrial fatty acid beta-oxidation network.. BMC Systems Biology.

[38] Klamt S, Grammel H, Straube R, Ghosh R, Gilles ED (2008). Modeling the electron transport chain of purple non-sulfur bacteria.. Molecular Systems Biology.

[39] Fato R, Estornell E, Di Bernardo S, Pallotti F, Parenti Castelli G (1996). Steady-state kinetics of the reduction of coenzyme Q analogs by complex I (NADH: ubiquinone oxidoreductase) in bovine heart mitochondria and submitochondrial particles.. Biochemistry.

[40] Stryer L, Biochemistry W (1995). Freeman and Company..

[41] Beard DA, Qian H (2008). Chemical biophysics: quantitative analysis of cellular systems..

[42] Hill T (1977). Free Energy Transduction in Biology..

[43] Hill T (1989). Free energy transduction and biochemical cycle kinetics..

[44] Chen X, Qi F, Dash R, Beard D (2010). Kinetics and regulation of mammalian NADH-ubiquinone oxidoreductase (Complex I).. Biophysical journal.

[45] Cleland W (1963). The kinetics of enzyme-catalyzed reactions with two or more substrates or products:: I. Nomenclature and rate equations.. Biochimica et Biophysica Acta (BBA)-Specialized Section on Enzymological Subjects.

[46] Marangoni A (2003). Enzyme kinetics: a modern approach..

[47] Nelson N, Sacher A, Nelson H (2002). Opinion: The significance of molecular slips in transport systems.. Nature Reviews Molecular Cell Biology.

[48] Onsager L (1931). Reciprocal relations in irreversible processes. i.. Phys Rev.

[49] Kacser H, Burns J (1973). The control of ux.. Symposia of the Society for Experimental Biology.

[50] Rossignol R, Malgat M, Mazat JP, Letellier T (1999). Threshold effect and tissue specificity.. Journal of Biological Chemistry.

